# Depression in Chinese adolescents: prevalence, symptoms, risk factors, affected brain, diagnosis, and interventions

**DOI:** 10.3389/fpsyg.2026.1816161

**Published:** 2026-08-07

**Authors:** Abigail Yoonbin Yang, Yinxuan Zhang, Sungchil Yang

**Affiliations:** 1 International Christian School, Sha Tin, Hong Kong SAR, China; 2 Department of Neuroscience, City University of Hong Kong, Kowloon, Hong Kong SAR, China

**Keywords:** diseased brain, mental disorders, psychiatry, public health, young mind

## Abstract

Major depressive disorder (MDD) has emerged as an urgent global public health concern with a pronounced impact on adolescents. Despite heightened awareness and advancements in diagnostic and therapeutic approaches, depression among Chinese adolescents remains highly prevalent. Furthermore, evidence suggests that this burden continues to increase amid China’s rapid urbanization and industrialization. Depression frequently emerges during adolescence and, if left untreated, may persist into adulthood and lead to long-term adverse outcomes. This review examines the current state of research on depression among Chinese adolescents, highlighting that precise identification of the disorder’s presence and etiology is essential for the development of effective diagnostic and therapeutic strategies. Furthermore, it explores the prevalence and risk factors associated with adolescent depression, elucidates the neural substrates implicated in the disorder, and evaluates existing diagnostic and therapeutic methodologies. Overall, this review highlights the importance of the timely recognition of risk factors and underlying mechanisms to support more effective prevention and treatment strategies in China.

## Introduction

Major depressive disorder (MDD) is a widespread mental health condition that is increasing, causing alarm in modern societies. This mental disorder has a complex etiology involving genetic, institutional, and environmental factors, and a multifactorial presentation characterized by sadness, irritability, social isolation, and psychophysiological changes, such as appetite loss and anhedonia (Belmaker and Agam, 2008; Langlieb and DePaulo, 2008). Approximately 280 million people worldwide are affected by MDD, and the prevalence continues to increase every year (Disease et al., 2018). In China, depression has elicited tremendous attention owing to the unpredictable increases in treatment-related societal and economic burdens in recent years. Increasing access to information and mental health awareness has enabled the public to become more informed about the physico-psychological hazards of depression, particularly among adolescents.

Recently, China’s National Health Commission has emphasized the importance of improving early diagnosis and targeted interventions for high-risk groups, identifying adolescents as one of the most vulnerable populations. In China, more than 20% of adolescents are diagnosed with depression, consistent with a meta-analysis reporting an overall prevalence consistently over 20% among children and adolescents, with regional variations (Zhou et al., 2024). These statistics highlight the concerning state of adolescent mental health in China and the urgent need for effective intervention and treatment strategies. Early diagnostic and therapeutic methods for monitoring and alleviating depressive symptoms in adolescents are crucial. In this review, we present results from meta-analyses of the prevalence and social impact of depression and related risk factors, discuss the main neurological and neuroanatomical factors involved, and suggest strategic plans for effective diagnosis and treatment.

## Prevalence of adolescent depression

### Prevalence among Chinese adolescents

Recent meta-analyses have estimated that approximately 21.3% of adolescents worldwide experience mild to severe depression, 18.9% suffer from moderate-to-severe forms, and 3.7% meet the criteria for MDD (Korczak et al., 2023; Feng and Wang, 2025). According to a report by the World Health Organization, depression and anxiety are among the leading causes of illness-related disability among adolescents aged 15–19 years worldwide, and suicide is one of the main causes of death in this population. Differences in how depression is defined, diagnosed, and measured across epidemiological studies have led to differences in statistics across countries and regions (Disease et al., 2018). In China, as in many other Asian countries, mental health disorders such as depression are stigmatized. This social stigma discourages individuals from seeking professional counsel, resulting in low reported rates of depression. Individuals who were previously diagnosed with depressive symptoms can often present with more severe symptoms later in life (He et al., 2021).

A comprehensive meta-analysis estimated a pooled prevalence of depressive symptoms of 23.5% among Chinese children and adolescents aged 7–18 years (Feng and Wang, 2025). Another meta-analysis of individuals aged 6–24 reported a slightly higher prevalence of 26.17% (Zhou et al., 2024), suggesting that adolescence is a determinant period for the induction of depression. Early-life stress has been identified as an important risk factor for the development of emotional and mental illnesses (Li F. et al., 2022). Comparisons of data from different time periods have consistently shown an upward trend in the prevalence of adolescent depression. A meta-analysis covering the 2000–2014 period reported a prevalence rate of 15.4%, while a more recent meta-analysis covering 2015–2024 reported a significantly higher rate of 23.3% (Feng and Wang, 2025). This rising prevalence of adolescent depression may be attributable to both the COVID-19 pandemic and increasing mental health awareness (Chen et al., 2022; Feng and Wang, 2025; Zhou et al., 2024). Since the pandemic, adolescents’ daily lives and learning environments have been significantly disrupted. Quarantines, online classes, reduced physical activity, and increased screen time have had detrimental effects on these individuals’ mental health.

### Regional and demographic variations of adolescent depression in China

**Figure 1:**
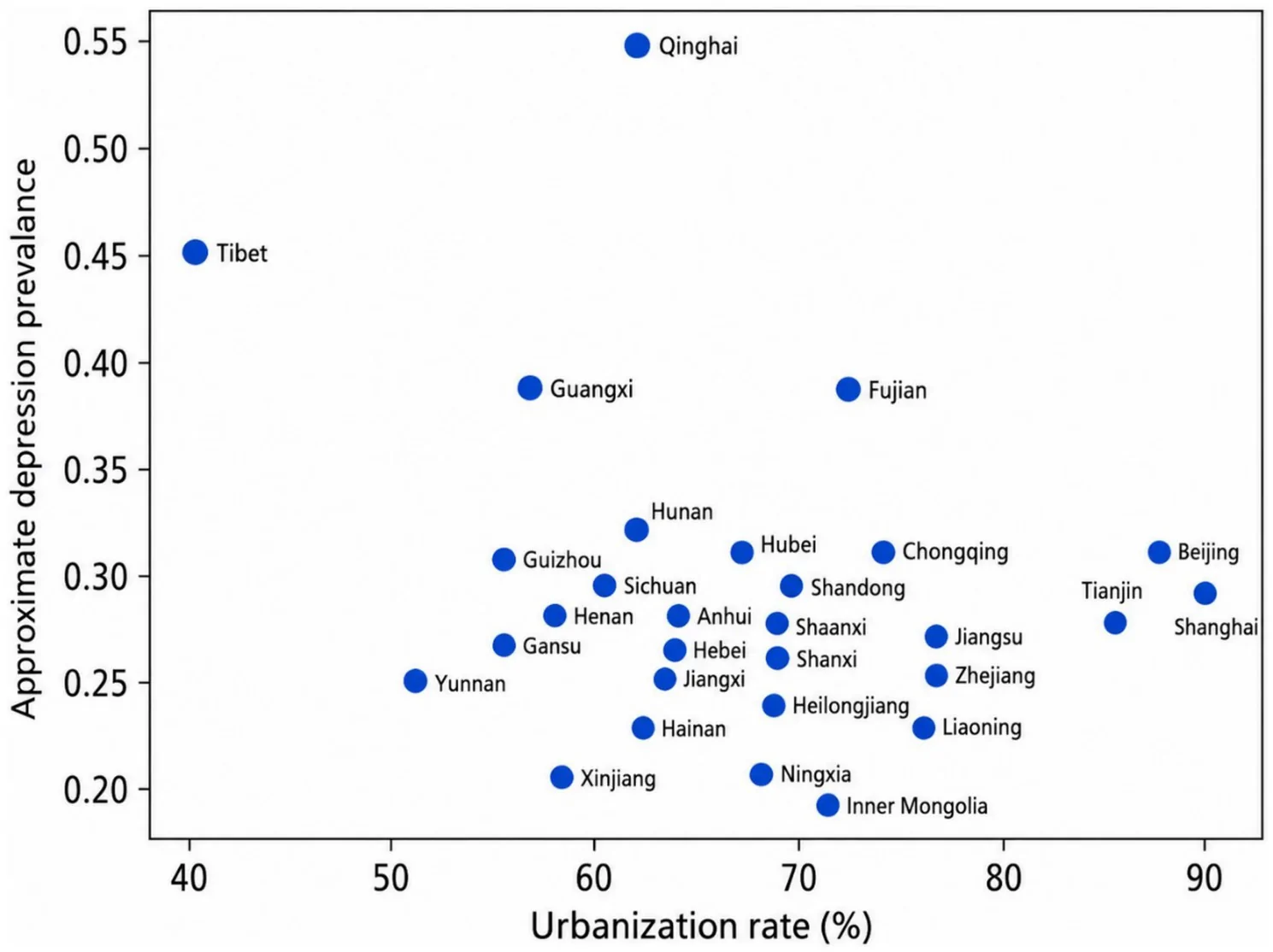
Prevalence of depression symptoms among children and adolescents and urbanization rate across Chinese provinces. Adapted from “Prevalence of depressive symptoms among children and adolescents in China: a systematic review and meta-analysis” and the National Bureau of Statistics of China. China Statistical Yearbook 2024 (China NBoSo, 2024; Zhou et al., 2024).

The prevalence of depression among Chinese children and adolescents shows substantial regional variation across China, although evidence regarding urban–rural differences remains inconsistent. Several studies have reported higher rates of depressive symptoms in rural areas than in urban areas (Jiang et al., 2022; Li W. Y. et al., 2022), which may reflect differences in socioeconomic conditions, educational opportunities, and access to social and mental health resources (Feng and Wang, 2025; Jiang et al., 2022). However, other studies have found limited urban–rural differences, suggesting that these patterns may vary according to factors such as age and study characteristics (Jiang et al., 2022). To further examine this relationship, prevalence estimates from a previous meta-analysis were combined with official provincial urbanization data and visualized in a scatter plot (Figure 1). Although provincial urbanization rates appear to trend with the prevalence of depressive symptoms among Chinese children and adolescents, no statistically significant association was observed between the two. This finding suggests that urbanization alone does not explain regional differences in adolescent depression and that multiple interacting factors—including socioeconomic, environmental, and demographic influences—are likely involved.

One factor that may contribute to the higher burden of depressive symptoms in some rural areas is the large population of left-behind children (LBC). In China, LBC are defined as individuals under 18 years of age who have been separated from one or both parents for at least 6 months, commonly due to parental migration for work (Jiang et al., 2022; Li et al., 2019). Previous studies suggest that prolonged parental absence may reduce emotional support and weaken parent–child relationships, increasing feelings of loneliness and emotional distress and thereby elevating the risk of depressive symptoms (Feng and Wang, 2025; Jiang et al., 2022).

Regional differences are also evident at the geographic level. A national meta-analysis reported the highest prevalence of adolescent depressive symptoms in eastern China (25.6%), followed by western (23.3%), northeastern (20.2%), and central regions (18.1%; Feng and Wang, 2025; Shorey et al., 2022). The relatively high prevalence observed in eastern China may partly reflect greater awareness of mental health issues and improved access to screening and services, which could increase detection rates (Feng and Wang, 2025). At the same time, adolescents in economically developed regions may experience greater academic and social pressures. In contrast, some high-altitude provinces, including Qinghai and Tibet, have reported prevalence estimates approaching 50% (Zhou et al., 2024), indicating that geographic and environmental contexts may also influence depression risk beyond levels of urbanization.

### Gender and age differences

Many studies have shown that, in China, the prevalence of depressive symptoms varies between boys and girls (Feng and Wang, 2025; Jiang et al., 2022; Li et al., 2019). In many regions and continents, such as the Middle East, Africa, and Asia, adolescent girls are generally more susceptible to depressive symptoms than boys (Shorey et al., 2022). However, some studies have reported only a small gender difference in depression detection rates; these discrepancies may be due to variations in the age ranges of the study samples (Tang et al., 2019; Zhou et al., 2024). Gender differences in depression typically begin to emerge during puberty, at around age 12, and increase through middle adolescence, peaking at around age 16 (Hankin and Abramson, 2001; Tang et al., 2019). During puberty, the unpredictable incidence of changes in female hormones may affect the homeostatic regulation of brain chemistry and lead to mood swings, thereby increasing the risk of depression among adolescent girls (Hankin and Abramson, 2001). Social disadvantages can also increase the probability of depressive symptoms among girls and women, who generally have a lower social status and fewer social resources than their male counterparts in many societies. Girls and women are vulnerable and exposed to gender-related traumas such as sexual abuse, harassment, lack of respect, limited life choices, and restricted access to leadership positions (Sun et al., 2023).

As aforementioned, age also has a significant influence on depression rates; in particular, the prevalence of depressive symptoms increases throughout adolescence. In China, high school students exhibit the highest levels of depressive symptoms, which are mostly attributable to intense academic pressure and high expectations (Jiang et al., 2022; Zhou et al., 2024). This pressure is closely linked to China’s highly competitive education system and becomes severe as students advance through school. Additionally, adolescents are vulnerable to stress and emotional instability due to the psychobiological changes that occur during this life stage (Lu et al., 2024). A mismatch between unstable hormonal levels and forced brain maturation to cope with social and cultural adaptation increases adolescents’ sensitivity to stress, especially among females (Zhou et al., 2024). As a result, senior high school students face an especially high risk of depression due to the combined impacts of high academic demands, emotional instability, and underdeveloped coping mechanisms. Taken together, these findings suggest that the symptoms, risk factors, diagnosis, and treatment of depression among Chinese adolescents should prudently consider the interaction between socioeconomical and biological factors.

## Symptoms of adolescent depression in China

The most common symptoms of adolescent depression include a self-perception of worthlessness, low energy, a sense of burdening others, and low mood. Related symptoms such as self-hatred, loneliness, sadness, pessimism, and isolation (Negele et al., 2015; Rice et al., 2019) are also widely observed. These symptoms can affect or be influenced largely by interpersonal interactions, especially as adolescence is a period of development in which people build deeper relationships and learn about themselves. Social interactions can significantly affect an individual’s self-perception and thus are related to intrapersonal symptoms of depression (Bernaras et al., 2019; Li X. et al., 2025). Group dynamics in which an individual perceives themselves as negligible can cause social stress and self-hatred, which ultimately may lead to isolation to avoid burdening others, with negative effects on reciprocal relationships (Garralda, 2025).

Cultural context also influences depressive symptomology. To date, only a few studies have investigated the specific symptoms of depressive adolescents in China, with a broad consensus that neurasthenia and somatization may be significant indicators of depression worldwide (Cheung et al., 2024). Family and interpersonal contexts may further influence depressive experiences. Traditional Chinese parenting practices have often been characterized as placing greater emphasis on correction and improvement than on explicit verbal praise. Consequently, adolescents who are exposed to persistent negative parental feedback may be more likely to develop negative self-perceptions and a reduced sense of self-worth, thereby increasing their vulnerability to depressive symptoms (Jiang et al., 2024). More broadly, Chinese culture has traditionally emphasized interdependence, fulfilling family responsibilities, and contributing to collective rather than individual goals (Tally, 2002). Within this context, adolescents may experience stronger pressure to meet familial and social expectations. When they perceive themselves as unable to contribute meaningfully or fulfill expected obligations, they may be more likely to experience feelings of guilt and self-criticism, which are commonly associated with depressive symptoms (Tally, 2002). In this context, interventions for Chinese adolescents with depression may benefit from incorporating these cultural perspectives by helping adolescents develop a stronger sense of self-worth, meaning, and belonging beyond perceived social or familial expectations.

**Figure 2:**
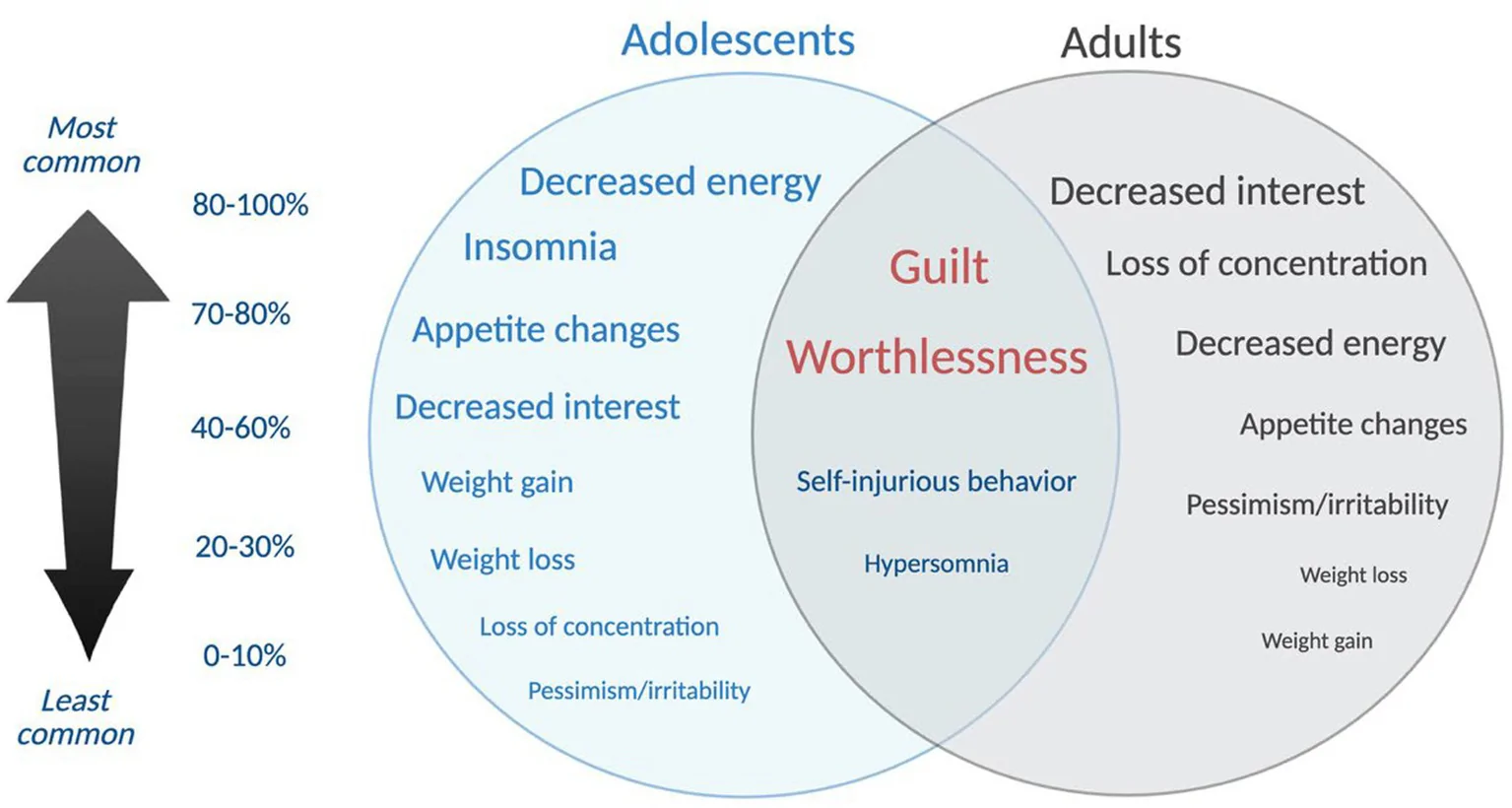
Comparison of symptoms of adolescent and adult depression. The blue circle lists symptoms of adolescent depression, while the gray circle lists symptoms of adult depression. The scale at left indicates the prevalence of common symptoms of depression in percentages. Larger words located higher within the circles indicate common symptoms, while smaller words with a lower location indicate less prevalent symptoms. Symptoms inside the cross section of the blue and gray circles indicate symptoms with a similar prevalence in both depressive adolescents and adults. The symptoms marked in red have been reported in Chinese adolescent subjects. Adapted from Rice et al. (2019); Jiang et al. (2024); and Tally (2002).

It is also worth noting that the clinical presentation of depression in adolescents differs from that observed in adults (Figure 2). In one study, families with both depressive parents and adolescents underwent symptom evaluations and sensitivity analysis (Rice et al., 2019). The most common symptoms among adolescents diagnosed with MDD were loss of energy, depressed mood, insomnia (sleep reduction by at least 2 h nightly), and feelings of worthlessness. Adolescents also reported a higher prevalence of vegetative symptoms (e.g., appetite and weight changes, loss of energy, and insomnia) than adults. Adolescents with vegetative symptoms had the highest rate of MDD (Rice et al., 2019). Conversely, adults most frequently displayed depressive mood, anhedonia, and feelings of worthlessness. They experienced loss of interest and decreased concentration more frequently than adolescents and did not exhibit prominent vegetative symptoms. However, adults and adolescents were found to share many symptoms, such as loss of energy, worthlessness, and depressive mood.

## Risk factors for depression among China adolescents

### Genetic factors

Heritability has been suggested to influence depression in adolescents, although heritability estimates vary widely (Rice, 2009; Rice, 2010). This variation is known to stem from differences in age, gender, symptoms, and measurement processes (Chen J. et al., 2014; Kwong et al., 2021; Rice, 2009). Regarding age, for instance, genetic effects on depression are negligible in childhood but increase during adolescence (Rice et al., 2002; Scourfield et al., 2003; Silberg et al., 1999; Thapar and McGuffin, 1994). In two contrasting China-specific studies of adolescent depression, one indicated a consequence of heritability (Chen J. et al., 2014), while another indicated no heritability (Unger et al., 2011). This discrepancy may be attributable to differences in the measurements used to test individual mood and behavior, as the studies used the Children’s Depression Inventory (CDI) and the Center for Epidemiologic Studies Depression Scale (CES-D), respectively (Chen J. et al., 2014). As CDI is considered to be more reliable for adolescents (Cole and Martin, 2005; Kenny and Zautra, 2001; Tram and Cole, 2006; Windle and Dumenci, 1998), one can conclude that heritability significantly affects depression in Chinese adolescents.

Gene–environment relationships are prominent in genetic studies of adolescent depression (Eaves et al., 2003; Jaffee and Price, 2007; Rice et al., 2003; Silberg et al., 1999). Gene–environment correlations can be passive, defined as when both the genes and environment are provided by an individual’s parents, and evocative, defined as when genes lead to certain behaviors that evoke responses from other people; they can also be active, defined as when genes propel an active search for certain environments (Jaffee and Price, 2007). Certain genes are linked to depression because they facilitate negative responses to stressful experiences (Eaves et al., 2003; Silberg et al., 1999; Thapar et al., 1998). Additionally, genetics determine individual’s responses to environmental stressors as measured gene–environment interactions (Moffitt et al., 2005). In the opposite direction, environmental factors can affect adolescents’ genetic liability for depression (Bronfenbrenner and Ceci, 1994; Hicks et al., 2009; Lau and Eley, 2008; Rice et al., 2006). These gene–environment connections suggest that the same genes could produce different results in Chinese and Western adolescents due to cultural differences (Greenberger et al., 2000b; Zhou et al., 2008). However, Chen H. D. et al. (2014) reported similar results between Chinese and Western adolescents in terms of the relationships of heritability and environmental factors with depression, suggesting that the general trends of relationships between the genetic and environmental factors that underlie adolescent depression applicable are across countries (Chen J. et al., 2014).

Definite conclusions about the molecular factors underlying the genetics of adolescent depression cannot be drawn due to insufficient information. However, trends have been observed for widely studied genes, such as the serotonin transporter-linked polymorphic region (5-HTTLPR) of the serotonin transporter gene and the Val66Met polymorphism in brain-derived neurotrophic factor (BDNF; Xia and Yao, 2015). 5-HTTLPR is associated with environmental stress and the risk of depression (Zwolinska et al., 2023) and has been shown to increase vulnerability to environmental stressors (Hankin et al., 2015; Jenness et al., 2011). Decreased BDNF synthesis has been correlated with an increased risk of adolescent depression (Bai et al., 2012; Youssef et al., 2018). This correlation may be indirect, however, as BDNF has been found to affect risk factors for depression such as suicide, cognitive impairment, and stress (Harrisberger et al., 2015; Hosang et al., 2014; Kimpton, 2012; Lockwood et al., 2015). Furthermore, the Val66Met polymorphism in the gene encoding BDNF appears to be associated with a liability for stressful events; the Val allele, rather than the Met allele, facilitates this liability (Chen et al., 2013; Hilt et al., 2007; Strauss et al., 2005; Zhao et al., 2018). Nonetheless, more research is needed to confirm the effects of 5-HTTLPR and Val66Met in Chinese adolescents. Other genes that have been associated with adolescent depression include protein-coding genes (e.g., those encoding multiple EGF-like domains 9 [*MEGF9*] and contactin-associated protein family member 3 [*CNTNAP3*]), genes encoding nuclear-localized ubiquitin-conjugating enzymes (e.g., *UBE2W* and *UBE2D1*), immune-responsive genes (e.g., the gene encoding FK506 binding protein 5 [*FKBP5*]), and genes encoding pro-inflammatory factors (e.g., interleukin-1 receptor accessary protein [IL1RAP], interleukin-1 beta [IL-1β], IL-6, tumor necrosis factor-alpha [TNF-*α*]), neurotrophic tyrosine kinase receptors and neuropeptides (e.g., cocaine and amphetamine regulated transcript [CART]), proteins associated with social behavior (e.g., oxytocin receptors), transcription factors (e.g., activator protein-2β [AP-2β]), and serotonin receptors (e.g., 5-hydroxytryptamine receptor 2A [5-HTR2A] and 5-HTR2C; Zhao et al., 2022). However, it is notable that recent large-scale studies have challenged the reproducibility of these findings, questioning the causal relationship of these genes with adolescent depression (Border et al., 2019; Vrshek-Schallhorn et al., 2019). While the molecular genetics of adolescent depression comprise a recently developed field, susceptibility to MDD is closely associated with a polygenetic trait involving interactions between genetic activity and environmental stressors (Mistry et al., 2018).

Another rising field of research, epigenetics in depression, suggests that epigenetics plays a crucial role in adolescent depression (Ochi and Dwivedi, 2023; Yuan et al., 2024; Zwolinska et al., 2023). For instance, *FKBP5* methylation levels have been linked to aspects of adolescent depression (Chiarella et al., 2020; Li W. Y. et al., 2022). Additionally, hypermethylation of the gene encoding the glucocorticoid nuclear receptor nuclear receptor subfamily 3 group C member 1 (*NR3C1*) is associated with substance use in adolescents (Raffetti et al., 2021) and depressive symptoms (Cicchetti and Handley, 2017; Gardini et al., 2022). *NR3C1* methylation was shown to precede MDD in adolescents (Humphreys et al., 2019). Increased methylation of the promoter of *SLC6A4*, which encodes a serotonin transporter, has been linked to adolescent depression and is probably caused by environmental stress (Ochi and Dwivedi, 2023; Swartz et al., 2017).

As mentioned in the previous section, gender differences in depression have been observed among Chinese adolescents. Although these differences increase during mid-adolescence, the age of their onset varies across countries mostly due to cultural differences and vulnerability to stressful life events, which are linked to genetic variation during maturation (Avenevoli et al., 2015; Ge et al., 1994; Greenberger et al., 2000a; Hankin and Abramson, 2001; Kessler and Üstün, 2004; Salk et al., 2017). Chinese adolescent girls were found to exhibit a greater risk of depression than boys because they exhibit an earlier increase in depressive symptoms (Avenevoli et al., 2015; Hankin et al., 1998; Salk et al., 2017). Psychological tendencies, physiological factors, and cultural norms also contribute to a greater risk of depression in girls than in boys (Chandra-Mouli et al., 2018; Cyranowski et al., 2000; Nolenhoeksema and Girgus, 1994; Schraedley et al., 1999; Sun et al., 2023). Specifically, girls may internalize their emotions, respond more sensitively to stress than boys, undergo gonadal hormone alterations during puberty, face traditional stereotypes of passivity and restricted choices, and receive a lack of respect, all of which can facilitate depressive symptoms (Angold et al., 1998; Cyranowski et al., 2000; Liu et al., 2022; Mcfarlane et al., 1994; Sun et al., 2023). In comparison, boys tend to externalize behaviors and have higher levels of self-esteem and confidence due to societal expectations of boldness and courage (Bleidorn et al., 2016; Chandra-Mouli et al., 2018; Mackenzie et al., 2006; Mcfarlane et al., 1994; Seedat et al., 2009). Due to China’s Confucian culture and socioeconomic system, sons are viewed as providing greater economic benefit than daughters, leading to a preference for sons among reproducing couples (Das Gupta et al., 2003; Wang X. J. et al., 2020); additionally, girls experience education inequality and low expectations (Sun et al., 2021).

### Environmental (or institutional) factors

Physical environmental factors such as noise, air pollution, and green spaces can influence adolescents’ mental well-being (Bezold et al., 2018; Hartley et al., 2021; Lian et al., 2023; Rudolph et al., 2019; Stansfeld and Matheson, 2003), particularly in China (Jiang et al., 2023; Ni et al., 2021; Xiang et al., 2022). Adolescents are more vulnerable to the environment than adults, as they are in a critical period of physical and mental development (Stansfeld and Matheson, 2003; Steinberg, 2005). Associations between increased noise and cognitive disruption have been studied in this age group (Basner et al., 2014; Rudolph et al., 2019; Schubert et al., 2019b). Noise is a ubiquitous element in urban cities, and the number of people exposed to environmental noise is expected to increase due to increasing urbanization (Buhaug and Urdal, 2013), industrialization, and the use of transportation worldwide (ITF, 2013). Several systematic reviews and meta-analyses have suggested close associations between environmental noise exposure and mental disorders such as depression (Eze et al., 2020; He et al., 2025; Hegewald et al., 2020; Schubert et al., 2019a), anxiety (He et al., 2025), and attention-deficit/hyperactivity disorder-related symptoms (Hegewald et al., 2020; Schubert et al., 2019a). In particular, adolescent depression is a strong risk factor for depressive disorders in adulthood (Heim and Binder, 2012; Pine et al., 1998).

Regarding other environmental factors, strong evidence suggests a positive relationship between ambient air pollution and depression in adolescents (Latham et al., 2021; Lian et al., 2023; Theron et al., 2022), which has been further supported by studies in China (Jiang et al., 2023; Li et al., 2024). Furthermore, green spaces are associated with improved mental and physical health in adolescents, and studies have shown that adolescents’ mood improves with exposure to nature (Chawla et al., 2014; Norwood et al., 2019; Tillmann et al., 2018; Vanaken and Danckaerts, 2018). Similarly, increased exposure to green areas has been correlated with decreased levels of depression in Chinese adolescents (Li et al., 2024). As mentioned above, however, rural adolescents in China have a higher risk of depression than their urban counterparts, despite living in rich green spaces. The primary reasons for this increased risk are a lack of mental health services and resources in rural areas (Kirby et al., 2019; Jiuling et al., 2016; Rao et al., 2019), as well as a lower awareness of depression and general health (Chen et al., 2019). In comparison, urban areas provide better living conditions, health information, and healthcare access, and thus more opportunities to take care of one’s mental health (Purtle et al., 2019; van der Wal et al., 2021; Yang et al., 2013). Discrepancies in socioeconomic status (SES) between urban and rural families are another factor, as lower family income has been correlated with an increased risk of depression in adolescents (Jiang et al., 2022; Reiss, 2013; Song and Tan, 2022; Zeng and Xu, 2024).

Academic stress leads to burnout and exhaustion and thus facilitates depression in adolescents (Jiang et al., 2021; Lin and Huang, 2014; May et al., 2015; Salmela-Aro and Upadyaya, 2014; Teuber et al., 2021). Supporting these observations, Chinese studies have shown that the risk of depression increases with the grade level in school (Ma et al., 2020; Zhang et al., 2021). China’s extremely rigorous education system, society, policies, and especially culture place great emphasis on scholastic achievement, thus increasing the centrality of academic pressure to the risk of depression (Jiang et al., 2021; Zhang et al., 2021). In China, education is considered the most crucial factor for monetary success and status (Hesketh and Ding, 2005; Li B. Q. et al., 2025). Furthermore, Confucian cultural values, which emphasize educational achievement, filial responsibility, and bringing honor to one’s family, contribute to the high academic expectations of many Asian parents (Chui and Wong, 2017). Accordingly, Chinese adolescents face great pressure to live up to their parents’ high academic expectations (Ang and Huan, 2006). This pressure has been exacerbated by the one-child policy in China, which have caused parental expectations and the pressure of representing the family to be focused on one child (Dello-Iacovo, 2009; Tsui and Rich, 2002). As Chinese adolescents thus prioritize education, academic pressure has become a primary risk factor for depression.

The associations of social media through the internet with adolescent depression have also been investigated. Spending more time online may increase the risk of depression by replacing protective psycho-emotionally rich activities, causing a lack of social involvement and a reduction in psychological well-being (Kim et al., 2009; Kong et al., 2023; Kraut et al., 1998). Although the adverse cognitive outcomes of heavy internet use often depend on genetic and environmental factors such as gender, parenting, and urbanization (Kong et al., 2023; Zhou and Ding, 2023), a general consensus has been reached regarding a direct relationship between social media addiction and adolescent depression (Lin et al., 2016; Nesi and Prinstein, 2015; Steers et al., 2014). This relationship is mediated by negative upward comparison, promoted by social networking sites that allow individuals to idealize themselves through posts and provide easy exposure to others’ lives, leading to social comparison, envy, and negative self-portrayals (Chou and Edge, 2012; Feinstein et al., 2014; Kim and Chock, 2015; Lin and Utz, 2015; Steers et al., 2014; Vogel et al., 2015). Adolescence marks a critical time of substantial cognitive and emotional development, and thus adolescents may be more susceptible to these negative effects than those of other ages (Cunningham et al., 2021; Shapiro and Margolin, 2014).

Stigma, or negative assumptions, about the nature of people with mental illnesses prevents help-seeking and is a risk factor for depressive symptoms with a high prevalence in China (Angermeyer et al., 2017; Boerema et al., 2016; Chen H. D. et al., 2014; Chen et al., 2016; Link and Phelan, 2006; Liu et al., 2016; Livingston and Boyd, 2010). The public is generally unaware about depression and the availability of professional mental health services (Huang et al., 2019; Huang et al., 2025; Yang et al., 2020). China’s Confucian culture also supports the belief that addressing one’s mental issues privately is more beneficial and displays less weakness of character than seeking assistance (Chen H. D. et al., 2014; Griffiths et al., 2011; Lam et al., 2010). Interpersonal harmony to maintain a good individual image in one’s family and community is also emphasized in China (Huang, 2016; Triandis, 1989; Yang et al., 2020). Relationship harmony is a central part of Chinese culture, and accordingly, the family is an essential unit in China with a major impact on adolescent development (Kavikondala et al., 2016; Lansford et al., 2018; Liu et al., 2019; Wang Y. H. et al., 2020). Societal stigmas often cause negative self-perception, which increases depressive symptoms such as shame while reducing self-esteem and self-efficacy (Garland and Zigler, 1994; SchonertReichl and Muller, 1996).

Positive parent–child communication and parental support are protective factors, whereas an adverse relationship is a risk factor for adolescent depression (Chang et al., 2018; Liu et al., 2019; Ma et al., 2020; Tang et al., 2020). Chinese parents tend to be more authoritarian and less expressive, while children are encouraged to be obedient and respectful and to exhibit self-control (Chao, 1994; Liu et al., 2005; Ma et al., 2020; Wang and Ollendick, 2001; Wu et al., 2002). As adolescents begin to exert more independence with age, these protective factors become less effective in relieving mood disorders (Li et al., 2015; Zhang et al., 2021). Furthermore, academic pressure, exacerbated by the one-child policy, places increased pressure on adolescents (Liu et al., 2019; Zhang et al., 2021). Inter-parental relationships similarly affect adolescents’ mental health (Li et al., 2020).

### Psychological factors

Although environmental and institutional factors shape the broader context in which adolescents develop, these influences may ultimately affect depression through their impact on psychological processes, including cognitive factors, personality traits, and coping mechanisms, are a major cause of depression in adolescents. Many studies have identified correlations of resilience, rumination, and personality traits with adolescent MDD. Resilience, defined as the ability to endure in the face of negative events, is widely acknowledged as a major contributor to adolescent depression and, when positively utilized, as a reliable protective and compensatory factor for adolescent depression in general (Davydov et al., 2010; Hjemdal et al., 2011; Luthar et al., 2000; Wingo et al., 2010), as well as in China (Chung et al., 2020; Gong et al., 2020; Liu et al., 2023; Liu et al., 2019; Tang et al., 2020). Resilience not only promotes other protective factors, such as self-esteem, social support, coping strategies, and positive emotions, but also guards against adverse environmental factors, such as stress or childhood trauma, thus counteracting psychological risk factors for depression such as negative emotions, rumination, or neuroticism (Anyan and Hjemdal, 2016; Connor and Davidson, 2003; Guo et al., 2024; Liu et al., 2023; Liu et al., 2017; Niu et al., 2016; Schulz et al., 2014; Wingo et al., 2010). Rumination, which is characterized by repetitive reflections about depressive symptoms to resolve them, has been identified as a risk factor for adolescent depression both internationally (Guo et al., 2024; Treynor et al., 2003) and in China (Chu et al., 2019; Hong et al., 2010; Liu et al., 2023; Wang et al., 2024), especially when coupled with negative life events. Rumination creates a self-exacerbating cycle that depletes a limited supply of mental resources, leading to compulsivity and depressive symptoms (Koster et al., 2011; Kraft et al., 2017; Stange et al., 2017). Personality traits can be categorized as a group of unchanging characteristics that contribute to an individual’s regular behavior. The Big Five personality traits, namely neuroticism, conscientiousness, agreeableness, extraversion, and openness, have been studied most widely in adolescent depression (Digman, 1997; Lyon et al., 2020; Markon et al., 2005). Neuroticism, defined as an inclination to respond negatively to adverse events, has been identified as a strong risk factor for depression in general (Kendler et al., 2004; Lahey, 2009; Smith et al., 2018), as well as in China (Gong et al., 2020; Tang et al., 2020; Wang et al., 2025; Yang et al., 2023). It is positively correlated with rumination, low self-esteem, perfectionism, and irritability (Castagna, 2019; Hughes et al., 2020; Smith et al., 2018). Extraversion, agreeableness, and conscientiousness have been identified as protective factors against depression in Chinese and other adolescents (Gong et al., 2020; Wang et al., 2025; Wu et al., 2024; Yang et al., 2023). Extraversion is especially important with respect to depression in adolescents because of their increased social vulnerability, and it has been observed to improve physical well-being (Diener et al., 2003; Hakulinen et al., 2015; McVarnock and Closson, 2022; Wang et al., 2025).

Self-esteem, defined as a person’s perception of themselves, is also correlated with depression in adolescents, including those in China (Campbell et al., 1991; Méndez et al., 2020; Sowislo and Orth, 2013; Wang X. C. et al. 2020). This trait impacts both individual factors (e.g., stability and positivity) and social interactions (Bondü et al., 2017; Campbell et al., 1991; Norona et al., 2016). A study of the main forms of self-esteem correlated with depression in Chinese adolescents identified social confidence, learning capacity, and physical appearance (Zhou et al., 2020), the last of which is due to Chinese culture (Chen et al., 2006; Jackson and Chen, 2008). Similarly, self-efficacy, an individual’s belief in their ability to achieve and control certain outcomes, has been negatively correlated with depression in adolescents internationally (Hankin, 2008; Kim et al., 2019; Shorey et al., 2022), as well as in China (Chang et al., 2018; Lin et al., 2024; Liu et al., 2024). Studies have suggested that the correlations of self-esteem and self-efficacy with adolescent depression may be dampened in collectivist countries such as China, which emphasize relationship harmony over personal accomplishment (Chang et al., 2018; Orth and Robins, 2013). However, many studies involving Chinese depressive adolescents have supported the importance of these psychological factors, which reflect cultural and socioeconomic changes in China (Parker et al., 2001).

## Brain areas involved in adolescent depression

**Figure 3:**
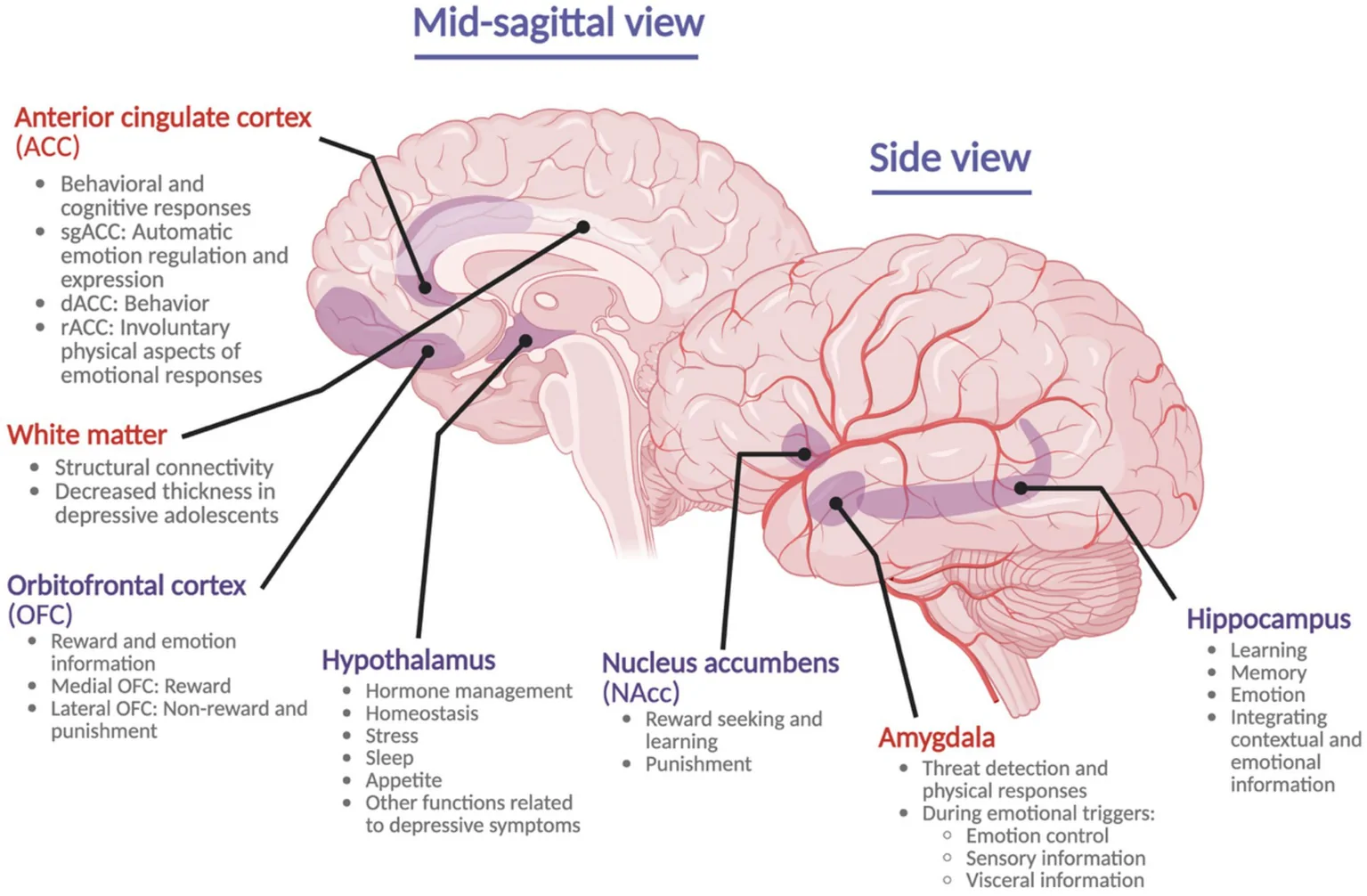
Brain areas relevant to adolescent depression. Purple or white highlights and black arrows indicate the location of the brain areas correlated to depression. The brain areas marked in red have been studied in Chinese adolescent subjects. A short summary of how the brain area contributions to adolescent depression is denoted. Adapted from Rolls et al. (2020); Lichenstein et al. (2016); Cullen et al. (2014a); Schriber et al. (2017); Lee et al. (2020); Guo et al. (2024); Goel et al. (2024); and Henderson et al. (2013).

During adolescence, neutral structures and systems undergo considerable development (Andersen, 2003; Casey, 2015; Casey et al., 2008). In particular, adolescents become more conscious of social contexts (Casey et al., 2008; Crone and Dahl, 2012; Schriber and Guyer, 2016). The affective network, which includes the orbitofrontal cortex (OFC), anterior cingulate cortex (ACC), amygdala, hippocampus, hypothalamus, and nucleus accumbens, has been implicated in depression. These network areas are interconnected and responsible for psycho-emotional processing and motivated behaviors involving the modulation of serotonergic and dopaminergic innervations (Belmaker and Agam, 2008; Delva and Stanwood, 2021; Pandya et al., 2012; Vahid-Ansari and Albert, 2021). Depressed adolescents exhibit abnormal activity in these regions, particularly when performing tasks that demand emotional regulation and cognitive control (Figure 3). Although other brain regions are also modulated by depression, these widely studied regions are highlighted here. A deeper understanding of the development and functioning of these neural circuits, as well as their relationship with adolescent depression, may contribute to the development of more targeted and effective prevention and treatment strategies.

### Orbitofrontal cortex

The OFC, located on the ventral surface of the frontal lobe, plays a key role in managing reward and emotion information. The medial OFC detects rewards, while the lateral OFC measures non-rewards and punishment (Rolls et al., 2020). Excessive activity in the lateral OFC is correlated with the severity of depressive symptoms in both adolescents and adults, suggesting its involvement in the development of depression (Xie et al., 2021). Remarkably, the cytosolic choline level in the OFC and irregular processing of error signals increase during the period of depression, especially when coupled with a family history of depression, thus demonstrating increased MDD susceptibility in adolescents (Jin et al., 2017; Steingard et al., 2000; Willinger et al., 2022). Accordingly, damage to the OFC elicits emotional instability, abnormal responses, social misbehavior, and depression symptoms (Jin et al., 2017).

### Anterior cingulate cortex

The ACC is situated within the cingulum bundle, which oversees communication concerning subcortical structures such as the striatum, hippocampus, sensorimotor cortices, and anterior thalamic radiations. As the center of information that regulates behavioral and cognitive responses such as valuation, performance monitoring, pain control, and the representation of requirements, this region is suggested to play an influential role in adolescent psychopathology (Chang and Isoda, 2013; Gasquoine, 2013; Lichenstein et al., 2016; Shackman et al., 2011; Shenhav et al., 2013; Weston, 2012). Structurally, the ACC can be divided into three sections: subgenual ACC (sgACC), dorsal ACC (dACC), and rostral ACC (rACC). The sgACC enables the automatic management and expression of emotions in response to different situations and thus may be connected to the negative emotional systems of depressive adolescents (Drevets and Savitz, 2008; Lichenstein et al., 2016; Phillips et al., 2008). The dACC is part of the salience network that conducts goal-directed behavior; this section determines actions by referring to previously instilled information and coordinating with motor regions (Rive et al., 2013; Shackman et al., 2011). Finally, the rACC is theorized to operate the involuntary physical components of emotional responses, such as autonomic, visceromotor, and endocrine reactions, and is connected to both the sgACC and dACC (Sturm et al., 2013). The rACC is also linked to the default mode network (DMN), which assists self-referential thinking and social cognitive functions (Fair et al., 2008; Lichenstein et al., 2016; Northoff, 2014). Severe adolescent psychopathy is linked to abnormally high connectivity and activity in the DMN, suggesting that the rACC directly impacts adolescent depression (Lichenstein et al., 2016). In summary, irregular functional and structural connectivity of the ACC disrupts emotional self-regulation, suggesting that this region is crucial for adolescent depression (Lichenstein et al., 2016).

### Amygdala

The amygdala is a key brain region that processes threats and arranges emotional and physiological reactions (LeDoux, 2000). It also contributes to depression-related functions such as emotion control, sensory information, and visceral information in the context of emotional triggers (Drevets and Savitz, 2008; Price, 2003). Studies of MDD in adolescents have highlighted abnormal resting state functional connectivity (RSFC) of the amygdala (Connolly et al., 2017; Cullen et al., 2014a; Tang et al., 2018). A dysfunctional amygdala has been correlated with cognitive impairment, emotional dysregulation, negative rumination, and vegetative characteristics of MDD (Gogtay et al., 2004; Kerestes et al., 2014; Perlman et al., 2012; Tang et al., 2018). Accordingly, depressed adolescents have perturbed amygdalar function, display aberrant RSFC with the amygdala and ACC networks, and exhibit decreased and positive RSFC with the hippocampus and parahippocampus, as well as decreased connectivity with some prefrontal cortices and the insula (Beesdo et al., 2009; Connolly et al., 2017; Cullen et al., 2014b; Hulvershorn et al., 2011; Pannekoek et al., 2014; Perlman et al., 2012; Roberson-Nay et al., 2006). Decreased functional and structural connectivity between the left amygdala and left ventral PFC was also observed in Chinese depressed adolescents (Wu et al., 2019).

### Hippocampus

The hippocampus, an essential part of the limbic system, is firmly connected to various brain regions, such as the amygdala, nucleus accumbens, and prefrontal cortex, that contribute to motivation and affective processing (Choi et al., 2021; Eichenbaum, 2013; Fastenrath et al., 2014; Thierry et al., 2000). This region is involved in learning, memory, and emotional regulation and the synthesis of contextual and emotional information (Eichenbaum et al., 2007; Pak et al., 2022a; Phillips et al., 2003; Seminowicz et al., 2004). The hippocampus is associated with MDD because of its connectome and function (Pak et al., 2022b; Schriber et al., 2017; Seminowicz et al., 2004). Abnormalities in hippocampal volume have been observed in adolescents with depression, while decreased hippocampal volume is commonly observed among depressive adults (Bremner et al., 2000; Schriber et al., 2017; Videbech and Ravnkilde, 2004). However, the relationship between hippocampal volume and depression in adolescents remains controversial, with studies reporting decreases (Koolschijn et al., 2013; Rao et al., 2010; Schriber et al., 2017), increases (Martinez et al., 2024), or no changes in volume (Rosso et al., 2005; Schriber et al., 2017; Yap et al., 2008), suggesting that hippocampal volume is only one of several functional and anatomical measures. From the perspective of brain lateralization, most studies indicate that the left side of the hippocampus plays a greater role than the right (Bremner et al., 2000; Martinez et al., 2024; Vythilingam et al., 2002). Additionally, decreased RSFC between the hippocampus and prefrontal cortex is related to irregular emotional and cognitive function and thus to adolescent depression (Geng et al., 2016).

### Nucleus accumbens

Within the ventral striatum, the NAc is a limbic structure laden with dopamine receptors, which can both promote motivation and decipher punishment and pain-related information (Harris and Peng, 2020; Ikemoto and Panksepp, 1999). NAc activity elicits motivated reward seeking and learning behavior, suggesting that it may be involved in adolescent depression (Daniel and Pollmann, 2014; Goff et al., 2013; Stringaris et al., 2015; Ye et al., 2023). In general, dulled reactivity in the ventral striatum negatively influences motivation processing and reduces reward anticipation and thus is related to early-life stress and depression severity in adolescents (Goff et al., 2013; Olino et al., 2014; Rappaport et al., 2020). Hyporeactivity of the NAc only upon reward prediction is correlated with depression, particularly upon the receipt of a reward (Auerbach et al., 2022; Keren et al., 2018; Olino et al., 2014; Rappaport et al., 2020; Stringaris et al., 2015). Additionally, changes in the NAc volume have been reported in depressive adolescents (Lee et al., 2020).

### Hypothalamus

The hypothalamus is a significant intermediary between the nervous and endocrine systems, with implications for hormone management, homeostasis, stress, sleep, appetite, and other bodily processes that can be related to depressive symptomology in adults (Goel et al., 2024). Hormonal commands are delivered along the hypothalamic–pituitary–adrenal (HPA) axis, which is heavily involved in managing the release of cortisol, a stress-related metabolic hormone (Guerry and Hastings, 2011; Keller et al., 2017). HPA dysregulation leads to altered cortisol levels in response to environmental and psychological stress, indicating an important role for the HPA axis in adolescent depression (Guerry and Hastings, 2011; Lopez-Duran et al., 2009). For example, irregularly lengthy periods of elevated cortisol are harmful to the body and can induce depressive symptoms (Guerry and Hastings, 2011; Gurguis et al., 1990).

### The white and gray matter in volume

The white matter pathway is crucial for structural connectivity in depression. Weakened white matter integrity, particularly in the corpus callosum, anterior thalamic radiation, anterior cingulum, and sagittal stratum, has been associated with MDD in adolescents (Henderson et al., 2013; Lichenstein et al., 2016). Gray matter structural abnormalities have been found in the lingual gyrus, middle occipital gyrus, postcentral gyrus, precentral gyrus, vermis, frontal–temporal–parietal regions, and subcortical regions of adolescents with depression (Mao et al., 2020; Zhang et al., 2023). Decreased cortical thickness in visual, auditory, and motor-related brain regions also has observed in this population (Zhang et al., 2023).

### Brain areas for Chinese adolescents with depression

A few studies using Chinese adolescent subjects have related depression with certain brain areas (Figure 3). Higher spontaneous resting-state activity of neurons in resting-state functional magnetic resonance imaging was found in the anterior and posterior cingulate gyrus, left inferior temporal gyrus, right superior temporal gyrus, right insula, right parietal lobe, and right fusiform gyrus, and lower activity in the bilateral cuneus, the left occipital lobe, and the left medial frontal lobe (Gong et al., 2014; Xiong et al., 2025). Also, abnormal cerebral gray matter in the lingual gyrus, middle occipital gyrus, postcentral gyrus, precentral gyrus, and vermis were observed in Chinese adolescents who suffer from depression (Mao et al., 2020). The symptom of depression is involved in sensory deficits, such as lower cortical thickness in visual processing, auditory processing, and motor movement brain areas (Xiong et al., 2025). Functional abnormalities coincide with structural changes, such as connectivity between the left amygdala and left ventral PFC (Wu et al., 2019). Although anatomical studies with Chinese adolescents have not been scrutinized at a specific brain area in identifying the cause and consequence of depression, there is a consensus that depression affects large brain areas, making it intractable and insidious for treatment.

## Diagnoses and assessments of depression in Chinese adolescents

As discussed above, depression in children and adolescents arises from complex interactions among genetic, neurobiological, cognitive, interpersonal, and environmental factors across development. Gene–environment interactions, developmental timing, and individual differences in personality, cognitive style, and coping strategies may further influence vulnerability to depressive symptoms (Uma Rao L-AC, 2009). This multifactorial and developmental nature of adolescent depression presents challenges for clinical identification. Therefore, accurate diagnosis and comprehensive assessment are essential for timely intervention and effective treatment. However, adolescence is a period of vibrant physical and psychological changes, which make the diagnosis of depression complex and challenging (Korczak et al., 2023; Liu et al., 2025).

### Screening for adolescent depression

Standardized guidelines have been developed for the screening and assessment of adolescent depression (Table 1). These guidelines are intended for use as assessment tools to effectively identify depressive symptoms in children and adolescents. For example, the U. S. Preventive Services Task Force (USPSTF) advises that all adolescents aged 12–18 years should be screened for MDD in primary care settings (Douglas et al., 2018). Such screening is supported by proper diagnosis, effective treatment, and post-hospital care (Selph and McDonagh, 2019). Many other screening tools are available for assessing adolescent depression, including the Hamilton Depression Scale, the Patient Health Questionnaire, the Beck Depression Inventory (BDI), the Center for Epidemiologic Studies Depression Scale (CES-D), the Children’s Depression Inventory (CDI), the Kutcher Adolescent Depression Scale, and the Reynolds Adolescent Depression Scale (Li B. Q. et al., 2025). Different screening tools are used to address different situations and emphases. To ensure validity and effectiveness, clinicians consider three key factors when selecting an assessment tool: the specific purpose of the assessment, the characteristics of the target population, and the available resources (Li X. et al., 2025). Specifically, the cultural background, which ranges from language and thinking patterns to culture-specific interpretations of emotional expression, plays an important role in how adolescents experience psycho-emotional disorders (Travaglia et al., 2018). For example, in East Asian cultures such as China and Japan, adolescents with depression are more likely to manifest their distress through somatic symptoms than by expressing negative emotions, which is discouraged by their culture (Li X. et al., 2025). However, as most existing screening scales for adolescent depression were developed within Western cultural contexts, they may not fully reflect how symptoms are expressed in Chinese adolescents. In China, self-report scales such as the BDI, CES-D, and CDI are currently the main tools used for depression screening. Although these measures have been shown to be useful for assessing Chinese adolescents, they are complicated by issues such as subjectivity and limited reliability (Feng and Wang, 2025). Therefore, there is an urgent need to improve screening guidelines optimized for Chinese adolescents with depression.

**Table 1:** Commonly used screening tools for adolescent depression.

Scale	Target population	Items	Key features	Citation
HAMD (Hamilton Depression Scale)	Clinician-rated, more prevalent in adolescents and adults	17/21/24 items	Widely used clinician-administered scales for assessing severity of depression symptoms	Li X. et al. (2025)
PHQ (Patient Health Questionnaire)	Self-report, adolescents and adults	2/9/15/8 items (PHQ-A for adolescents)	Widely used questionnaire to assess depression in adolescents	Douglas et al. (2018), Li B. Q. et al. (2025), Xuerong et al. (2024)
BDI (Beck Depression Inventory)	Self-report, adolescents and adults	21 items	Used to track depression severity and treatment progress via four key features: depressive mood, low self-esteem, physical symptoms, and social withdrawal	Li B. Q. et al. (2025), Xuerong et al. (2024)
ES-D (Center for Epidemiologic Studies Depression Scale)	Self-report, adolescents and adults	20 items	Designed for epidemiological studies; focuses on depressive mood frequency; effective for cross-sectional comparisons	Li B. Q. et al. (2025), Xuerong et al. (2024)
CDI (Children’s Depression Inventory)	Self-report, ages 7–17	27 items	Specifically designed for children and adolescents with five subscales: low self-esteem, negative mood, anhedonia, inefficiency, and interpersonal problems	Li B. Q. et al. (2025), Xuerong et al. (2024)
KADS (Kutcher Adolescent Depression Scale)	Self-report, adolescents	6/11/16 items	Designed to measure the frequency and severity of symptoms	Li X. et al. (2025)
RADS (Reynolds Adolescent Depression Scale)	Self-report, ages 12–18	30 items	Focuses on adolescents’ daily lives with four key features: mood, anhedonia, negative self-evaluation, and somatic complaints	Li X. et al. (2025)

Different screening tools are used in different situation and emphases according to age variation.

### Diagnosis of adolescent depression

A successful screening for adolescent depression should be followed by confirmation through a professional evaluation based on standardized diagnostic criteria (Chu et al., 2019). The two most commonly used diagnostic systems are the International Classification of Diseases (ICD) and the Diagnostic and Statistical Manual of Mental Disorders (DSM; Table 2; Bernaras et al., 2019; Shorey et al., 2022). In China, current recommendations emphasize that a diagnosis of depression should primarily follow the criteria specified in the ICD-11 and DSM-5(Garralda, 2025, Robles et al., 2022; Wang and Ji, 2023; Xuerong et al., 2024). The ICD-11 classifies depressive disorders within the category of mood disorders. Here, depressive disorders are defined as conditions characterized by a persistent depressed mood or loss of pleasure, accompanied by additional cognitive, behavioral, and neurovegetative symptoms that significantly impair an individual’s functioning (Garralda, 2025). In comparison, the DSM-5 requires at least five of nine specific symptoms to be present for a diagnosis of MDD, with at least one symptom being either a depressed mood or loss of interest/pleasure. Other symptoms include appetite or weight changes, sleep disturbances, psychomotor agitation or retardation, fatigue or loss of energy, diminished ability to think or concentrate, feelings of worthlessness or excessive guilt, and recurrent thoughts of death or suicidality (Tolentino and Schmidt, 2018). The accurate assessment and diagnosis of adolescent depression require culturally sensitive screening and a professional evaluation using standardized criteria, such as the ICD-11 or DSM-5, to ensure timely and effective intervention.

**Table 2:** Comparison of DSM-5 and ICD-11 criteria for depressive disorders.

Diagnostics	Classification	Diagnostic features/criteria	Citation
ICD-11 (International Classification of Diseases)	Depressive disorders	Depressed mood (e.g., sad, irritable, or empty) or loss of pleasure, accompanied by cognitive, behavioral, and neurovegetative symptoms that impair functioning; excludes individuals with manic, mixed, or hypomanic episodes.	Selph and McDonagh (2019); W. H. Organization (2026)
DSM-5 (Diagnostic and Statistical Manual of Mental Disorders)	Depressive disorders	At least five of nine symptoms during a 2-week period; one must be depressed mood or loss of interest/pleasure. Other symptoms include appetite or weight change, sleep disturbance, psychomotor changes, fatigue, concentration difficulties, worthlessness/guilt, and suicidality	Wang and Ji (2023)

## Treatments for adolescent depression

A meta-analysis revealed that the treatment rate for mental disorders among adolescents remains disappointingly low, particularly for conditions such as depression and anxiety (Wang et al., 2023). In that report, the overall treatment rate among adolescents was 36%, with variation according to region and income level. These findings highlight the importance of considering cultural and socioeconomic factors in the timely treatment of adolescent depression in China. Cultural background, religion, economic development, and national health policies help determine how easily adolescents can access mental health care and how mental illness is perceived and treated in their countries. Psychotherapy and pharmacological therapies are the most common treatments for depression (Ryan, 2005). However, many treatments were originally designed for adults and later adapted for use in adolescents (Maughan et al., 2013). As mentioned in the previous section, the causes and symptoms of depression in adults and adolescents are different; thus, it is important to select effective treatment choices solely for adolescents (Thapar et al., 2012).

### Pharmacotherapy

Pharmacotherapy is a common and effective method for alleviating depression. The most widely used antidepressant medications include selective serotonin reuptake inhibitors (SSRIs), selective serotonin–norepinephrine reuptake inhibitors (SNRIs), monoamine oxidase inhibitors (MAOIs), and tricyclic antidepressants (TCAs; Table 3; DeRubeis et al., 2008). However, research designed to test the efficacy of these medications in children and adolescents remains insufficient, and further confirmation and validation are needed, as the effectiveness of medication treatment for adolescent depression remains inconclusive (Jane Garland et al., 2016). Furthermore, adolescents’ neurotransmitter systems have not yet fully developed, and thus, they might not respond as effectively to antidepressant medications as adults (Wu et al., 2025). Decisions about medication treatment for adolescents must therefore be made with caution. In current clinical practice, SSRIs are considered the primary pharmacological treatment option for depression in adolescents (Yulong et al., 2022). SNRIs are generally not considered to be suitable as first-line treatment options (Jane Garland et al., 2016). Currently, SSRIs such as fluoxetine and escitalopram are the primary medications approved by the U. S. Food and Drug Administration (FDA) for treating depression in adolescents (Miller and Campo, 2021).

**Table 3:** Pharmacotherapy for adolescent depression. SSRIs are considered the primary pharmacological treatment option for depression in adolescents.

Drug class	Examples	Advantages	Limitations/side effects	Age/guidance	Citation
SSRIs (Selective serotonin reuptake inhibitors)	Fluoxetine, sertraline, escitalopram, fluvoxamin	First-line treatment for adolescents; approved by the U. S. FDA	Nausea, headache, fatigue, insomnia, risk of suicidal ideation in youth	Fluoxetine approved for ≥8 years; Escitalopram for ≥12 years	Duval et al. (2006); Hollon et al. (2002); Nemeroff (2006); Yulong et al. (2022)
SNRIs (Serotonin–norepinephrine reuptake inhibitors)	Venlafaxine, duloxetine	Comparable efficacy to SSRIs; used for comorbid pain/anxiety	Hypertension; risks of overdose and cardiac effects; limited evidence on efficacy and tolerability	Not recommended as first-line for adolescents	Duval et al. (2006); Nemeroff (2006)
MAOIs (Monoamine oxidase inhibitors)	Phenelzine, tranylcypromine isocarboxazid	Effective for “atypical” depression	Dry mouth, constipation, nausea, nervousness, insomnia, hypertensive crisis risk	Alternatives for patients unresponsive to standard treatments; rarely used in adolescents	Duval et al. (2006); Nemeroff (2006)
TCAs (Tricyclic antidepressants)	Amitriptyline, imipramine, nortriptyline clomipramin, doxepin	Low generic cost; effective for severe and melancholic depression	Anticholinergic side effects including dry mouth, blurred vision, constipation, urinary issues, weight gain; lethality in overdose	Initiated at low dosages; not preferred for adolescents	Hollon et al. (2002); Nemeroff (2006)

Several lines of evidence from randomized controlled trials and meta-analyses demonstrate the effectiveness, safety, and tolerability of fluoxetine for adolescent depression (Thapar et al., 2012; Yulong et al., 2022). This drug has been approved for use in children aged 8 years and older and is considered the most effective medication for treating acute moderate-to-severe depression in adolescents (Wang et al., 2023). Fluoxetine also exhibited effectiveness in terms of reducing the risk of relapse. A study of four adolescents demonstrated that treatment with fluoxetine over 6 months prevents additional relapse better than placebo (Jane Garland et al., 2016). While escitalopram was also effective, the FDA has approved this medication only for adolescents older than 12 years (Thapar et al., 2012; Wang et al., 2023). Common side effects of SSRIs and other antidepressant medications include headache, insomnia, fatigue, drowsiness, nausea, and vomiting (Braund et al., 2021; Ryan, 2005; Yulong et al., 2022). Furthermore, the U. S. FDA has issued a black-box warning, indicating that antidepressants may increase the risk of suicidal thoughts and behaviors in children and adolescents. In a meta-analysis, the U. S. FDA found a small but statistically significant increase in suicidal thoughts and behaviors among individuals taking antidepressants compared with those receiving a placebo. However, an increased risk of completed suicide has not been established (Mitchell et al., 2014), and evidence linking antidepressant use to overall suicide rates remains limited and inconclusive. Some studies have reported that, in most cases of adolescent suicide deaths, no detectable concentration of antidepressants was identified during autopsy, suggesting that medication noncompliance, rather than antidepressant treatment itself, may contribute to suicide attempts (Mitchell et al., 2014). Moreover, several studies have shown that suicide risk decreases with sustained antidepressant treatment, supporting the potential benefits of continued treatment (Naveed et al., 2021). Therefore, the relationship between antidepressant treatment and suicidality in youth remains complex and should be interpreted within a balanced risk–benefit framework, particularly in moderate to severe cases where untreated depression itself may increase the risk of functional impairment and suicidality. Evidence further suggests that close monitoring may be particularly important during the early phase of treatment initiation, when suicidal risk may be elevated in some individuals; accordingly, the FDA recommends careful monitoring of treatment response and emergent suicidality in patients receiving antidepressant therapy (Mitchell et al., 2014; Naveed et al., 2021).

In China, SSRIs remain the most commonly prescribed class of antidepressants. Sertraline holds the leading position, despite a continued decline in its usage rate. Fluoxetine, fluvoxamine, and escitalopram are the second, third, and fourth most used SSRIs, respectively (Wang and Ji, 2023). However, fluoxetine is the only treatment approved for treating moderate-to-severe depression in children (≥8 years). Notably, the choice of medication for adolescent depression in China depends on several factors, such as depression etiology, severity, age, clinical guidelines, clinical expertise, and market prices (Wang and Ji, 2023).

### Psychotherapy

Although medication treatment for adolescent depression has been proven effective, it remains limited in terms of long-lasting adherence and efficacy (Braund et al., 2021; Ryan, 2005). Antidepressant medications can effectively manage acute depressive episodes and prevent relapse during continued use. However, no evidence indicates that they reduce the risk of future relapse after discontinuation (DeRubeis et al., 2008). Psychotherapeutic treatment is another accepted intervention for depression with longer-lasting effects than pharmacotherapy, especially after treatment cessation (Cuijpers et al., 2019). In randomized trials, therapies are typically compared with control conditions such as care-as-usual, waiting lists, or placebo pills, and their therapeutic effects are generally effective compared with those of the control condition.

Cognitive behavioral therapy (CBT) and interpersonal therapy (IPT) can effectively treat adolescent depression and are currently used in clinical practice in China (Miller and Campo, 2021). No evidence is available regarding differences in the outcomes of various psychotherapeutic approaches for treating depression (Cuijpers et al., 2019). CBT is recommended for the treatment of adolescent depression by the American Psychological Association and National Institute for Health and Care Excellence (Dardas et al., 2023). CBT enables individuals to understand how negative thought patterns affect their emotions, behaviors, and overall mental health. It emphasizes the interactions between thoughts, feelings, and behaviors, aiming to reduce depressive symptoms and enhance coping abilities (Gautam et al., 2020; Miller and Campo, 2021). In practice, depressed adolescents are taught to recognize distorted or negative thoughts and restructure them into more realistic thoughts by evaluating the supporting and opposing evidence. As a result, CBT reduces not only emotional distress but also the risk of relapse (Dardas et al., 2023). For example, Chinese adolescents who frequently engage in rumination following academic setbacks or exposure to family expectations may repeatedly focus on perceived failures or negative self-evaluations. Through CBT, these adolescents can learn to identify such ruminative thought patterns and replace them with more balanced and realistic appraisals, thereby reducing the intensity and duration of depressive emotions. IPT focuses on the connection between depression and interpersonal relationships. It aims to reduce depressive symptoms by assisting patients in identifying the interpersonal triggers of their depression and addressing difficulties in their current relationships (Markowitz and Weissman, 2004; Miller and Campo, 2021). Through IPT, adolescent patients with depression learn to recognize and express their emotions, understand how their mood is related to their social interactions, and develop more effective communication and problem-solving skills.

### Combined treatment with psychotherapy and psychotherapy

In recent years, studies increasingly have shown that the combination of drug treatment and psychological therapy yields superior therapeutic effects for adolescent depression. According to the Treatment for Adolescents with Depression Study, combining CBT treatment with an SSRI resulted in a 71% response rate and was more effective than either CBT (43%) or placebo (35%) alone in two analyses; it also yielded advantages over SSRI (61%) alone in one of the analyses (Ryan, 2005). A recent study also found that combining CBT with medication was significantly more effective than either treatment alone (Dardas et al., 2023). Specifically, the measure used to compare treatment effectiveness showed that the combination therapy was 50% more effective in terms of producing short-term and long-term results than either CBT or medication alone. Overall, medication relieves depression by adjusting the actions of neurotransmitters in the nervous system, while psychotherapy supports recovery by helping patients change their negative thought patterns and build coping skills. Combined therapy can provide quick relief from symptoms and allows patients to develop long-term strategies to manage their emotions and prevent relapse, leading to more effective and longer-lasting results (Yuxin et al., 2025). A comprehensive treatment plan that includes both medication and psychotherapy is most effective for relieving symptoms and supporting long-term recovery in Chinese adolescents with depression (Wantong et al., 2025).

## Conclusion

The increasing prevalence and severe consequences of depression among Chinese adolescents underscore the urgent need for a comprehensive understanding of its contributing factors, neural mechanisms, and effective intervention strategies. Currently, the literature on adolescent depression in China remains highly fragmented—spanning disparate studies on risk factors, symptoms, and interventions—primarily due to a scarcity of comprehensive and holistic research focused exclusively on this demographic. Several other methodological limitations persist, including high heterogeneity among existing studies, inconsistent assessment tools, an incomplete understanding of sociocultural influences, and a lack of longitudinal and neurobiological data needed to establish causal relationships. Nonetheless, this review attempts to synthesize the existing evidence on the prevalence, clinical symptoms, risk factors, neural substrates, diagnostic approaches, and available treatments for depression in Chinese adolescents. By doing so, we aim to inform future prevention and intervention frameworks. Furthermore, we emphasize the critical need for early risk identification and targeted interventions to optimize clinical outcomes. Ultimately, enhancing early recognition, reducing stigma, expanding access to mental health resources, and deploying culturally tailored strategies will be vital for improving the psychological well-being and long-term social trajectories of adolescents in China.
